# More about Flogging

**Published:** 1921-04-02

**Authors:** 


**THE PUBLIC WELL-BEING**—(*continued*).

### More about Flogging.

### WHERE OPINION IS MISINFORMED.

A meeting of head masters and mistresses, specially called to discuss the question of corporal punishment, has carried on the interest in the article in a recent issue. The general sense of the meeting seemed to be that corporal punishment should be retained, while a line of cleavage almost amusing in its sharpness broke out between the heads of the public schools, who advocated flogging on the body, and the heads of the elementary schools, who were shocked at this and wished to continue to cane on the hand. They cannot both be right, unless it be argued that at a certain age a boy (and a girl, one might ask, though this delicate subject was not mentioned) graduates by process of time from the class which ought to be caned on the hand to the class which ought to be caned on the body.

With the too common attitude which classes all who object to corporal punishment as "sloppy sentimentalists" and those who advocate it as "inhuman brutes," we have nothing to do. The question cannot be discussed apart from sentiment,

THE PUBLIC WELL-BEING—(*continued*).

and it cannot be discussed profitably on the assumption that all those who advocate one view of it do so without good cause and motive. But it is, as a matter of fact, usually discussed without attention to two points of vital importance. One is the connection between flogging and sexual emotion. Dean Inge, for instance, has spoken of the "wholesomeness" of flogging, but Mr. Shaw declares that the desire to practise flogging is a real and mischievous disease. There is a connection between flogging and sexual emotion which may affect both the operator and his victim, and the forcible overcoming of the sufferer's sense of shame which some advocates of flogging regard as in itself useful has a dangerous possibility of breaking down other restraints due to shame. To those acquainted with an unpleasant side of a painful subject it is well known that flogging and submitting to being flogged are in fact a recognised method of commercial vice. It would be monstrous to deduce from this that even the strongest advocates of flogging are abnormally minded, or that sexual corruption is of necessity connected with flogging; but the light thrown by these facts on the nature of flogging and the use of corporal punishment as a means of education suggest that the whole subject demands not only clear thinking and right feeling, but a certain amount of exact knowledge in those who propose to put forward opinions on it.

The second point on which opinion is generally no less misinformed is the question of the efficacy of flogging. If flogging were proved to have the efficiency asserted by its advocates, that could not decide the question. The whole story of progress is the record of our capacity to abandon barbaric and out-of-date methods, because the time for them is past. Many savage methods would work to-day, but we have outgrown them, and for the ordinary man the question of their efficiency does not arise. Mr. Henry S. Salt, who was an Eton master, has gathered evidence to show that the notion of the efficiency of flogging which has indeed converted many opponents has no real basis. He quotes cases of criminals not only convicted again of the offence for which they were flogged, but flogged again within a short time. He points out that the argument that flogging is a deterrent is almost destroyed by the mere fact that some criminals ask to be flogged rather than be sent to gaol.

The outbreak of garrotting during the 'sixties of last century has been asserted again and again to have been put down by the lash, but the outbreak had been suppressed by the ordinary criminal law before the Flogging Bill could be got through Parliament; and two Home Secretaries, Mr. Asquith and Lord Ridley, have declared that the evil had been mastered before the Flogging Bill became law. As for flogging in our Dominions abroad, Sir Sidney Olivier, when Acting-Governor of Jamaica, said: "It is clear that the increased resort by magistrates to flogging in the case of second offenders is not checking it (prædial larceny) at the present time, and though floggings were being awarded nearly four times as frequently in the latter months of the year the tide was still rising, and only the return of plenty will check it."

The last clause is significant because of its general implication. Flogging is bad because it is an attempted short-cut. It seems a simple and effective remedy. As a matter of fact, it merely enables men to believe that evils are simple which, being complex, need subtle treatment. It sounds much more definite to have flogged a child than to have argued with him, and it is this specious simplicity which is the cause of the real popularity of flogging, but is, as a matter of fact, its strongest condemnation. There are other and better ways to achieve all the results even falsely claimed for flogging if men will take the trouble to employ them, and possess sufficient imagination.

